# Rational Targets of Therapy in Extranodal NK/T-Cell Lymphoma

**DOI:** 10.3390/cancers15051366

**Published:** 2023-02-21

**Authors:** Ajay Major, Pierluigi Porcu, Bradley M. Haverkos

**Affiliations:** 1 Division of Hematology, University of Colorado School of Medicine, Aurora, CO 80045, USA; 2 Division of Medical Oncology and Hematopoietic Stem Cell Transplantation, Thomas Jefferson University, Philadelphia, PA 19107, USA

**Keywords:** extranodal NK/T-cell lymphoma (ENKTL), biomarkers, novel therapies

## Abstract

**Simple Summary:**

Extranodal NK/T-cell lymphoma (ENKTL) is an aggressive blood cancer with poor survival rates, particularly for patients with advanced-stage and relapsed disease. Emerging research on the genetic and molecular causes of ENKTL have revealed new potential treatment strategies. In this review, we summarize how research on the biological causes of ENKTL has translated to new targets for the treatment of ENKTL, as well as the identification of new bi-omarkers which may predict prognosis and responses to specific anti-cancer therapies and enable a personalized medicine approach towards ENKTL therapy.

**Abstract:**

Extranodal NK/T-cell lymphoma (ENKTL) is an aggressive extranodal non-Hodgkin lymphoma (NHL) with poor outcomes, particularly in advanced-stage and relapsed/refractory disease. Emerging research on molecular drivers of ENKTL lymphomagenesis by next-generation and whole genome sequencing has revealed diverse genomic mutations in multiple signaling pathways, with the identification of multiple putative targets for novel therapeutic agents. In this review, we summarize the biological underpinnings of newly-understood therapeutic targets in ENKTL with a focus on translational implications, including epigenetic and histone regulatory aberrations, activation of cell proliferation signaling pathways, suppression of apoptosis and tumor suppressor genes, changes in the tumor microenvironment, and EBV-mediated oncogenesis. In addition, we highlight prognostic and predictive biomarkers which may enable a personalized medicine approach toward ENKTL therapy.

## 1. Background

Extranodal NK/T-cell lymphoma (ENKTL) is a rare extranodal non-Hodgkin lymphoma (NHL) that primarily occurs in the upper aerodigestive tract and has an aggressive presentation, with locoregional invasion in the nasopharynx causing necrosis, hemorrhage, and impingement on anatomic structures including the orbits [1,2,3]. The incidence of ENKTL is much higher in East Asia and Latin America than in Europe and North America, representing up to 15% of all NHL diagnoses, likely due to underlying geodemographic differences in human leukocyte antigen (HLA) genes and genetic susceptibility [4,5,6,7]. Outcomes for ENKTL are generally poor, with 5-year overall survival (OS) rates of approximately 50% when treated with asparaginase-based multi-agent combination chemotherapy regimens such as SMILE [8,9] but as low as 25–28% in high-risk patients as assessed by various prognostic indices of NK lymphoma [10,11]. The majority of patients with ENKTL present with limited-stage, localized disease, in which outcomes are better, with 5-year and 10-year OS rates of 64–89% and 57%, respectively, utilizing combination chemotherapy and radiation [12,13,14]. However, patients with relapsed or refractory disease have dismal outcomes, with median survival of less than 12 months [15,16]. Despite advances in the frontline management of ENKTL with combination chemoradiotherapy approaches [14], there is a pressing need for novel and rational therapies, informed by the molecular biology of ENKTL, particularly for patients with relapsed/refractory disease, for patients who cannot tolerate intensive frontline induction therapy, and for patients with high-risk disease features and advanced-stage disease.

## 2. Evolving Understanding of the Biology of ENKTL

Historically, the pathobiology of ENKTL and its molecular drivers have been poorly understood. Gene rearrangement studies suggest that the majority of ENKTL cases are of NK cell rather than T cell lineage [17,18,19]. Although there are no apparent differences in clinical presentation or survival between NK or T cell lineages [20], there is emerging evidence that different signaling pathways are constitutively activated in each cell lineage, reflecting a complex and heterogeneous molecular driver landscape of ENKTL [19]. Classical descriptions of ENKTL oncogenesis by canonical T and NK cell signaling have focused on the ubiquitous presence of EBV in ENKTL tumor cells, and it is posited that EBV infection induces the overexpression of the transmembrane oncoprotein LMP1 in infected NK and T cells, resulting in ligand-independent activation of the NF-κB and MAPK signaling pathways [18,19,21]. These signaling pathways, with loss of tumor suppressor genes on chromosome 6q21, as is commonly observed in ENKTL, cause tumor cell proliferation and avoidance of apoptosis by regulation of c-MYC and survivin and also upregulate cell surface expression of PD-L1 to promote escape from immune surveillance [18,22]. However, ENKTL cells do not experience immortalization in response to EBV infection, as is seen in B-cell lymphomas, but rather become sensitized to proliferative cytokines such as IL-2, suggesting that other molecular alterations must occur in a multistep process for EBV-infected NK or T cells to undergo malignant transformation [23,24]. Recent advances in somatic next-generation and whole-genome sequencing have provided emerging evidence that ENKTL is characterized by genomic aberrations in multiple signaling pathways, including broad epigenetic and gene methylation changes [17,19,23,25]. In this review, we summarize newly-understood putative driver mutations in ENKTL with a focus on translational implications and novel therapies with rational biologic underpinnings (Table 1), as well as prognostic and predictive biomarkers which may enable personalized therapeutic approaches (Table 2).

## 3. Epigenetic Aberrations in ENKTL

It is increasingly observed that epigenetic modifiers are the largest group of mutated genes in ENKTL, with one meta-analysis of nine next-generation sequencing (NGS) studies finding that 25% of identified mutations in ENKTL affected epigenetic regulators [19]. Epigenetic aberrations play a critical role in tumorigenesis by silencing tumor suppressor genes and altering the regulation of oncogenes. A cohesive understanding of how epigenetic changes drive the ENKTL clone is starting to emerge [32].

### 3.1. Promoter Hypermethylation

In many types of cancer, promoter regions of tumor suppressor genes are frequently hypermethylated during carcinogenesis, resulting in transcriptional silencing via the recruitment of histone deacetylases (HDACs) and repressive chromatin formation [32]. Comparison of ENKTL tumor cells and normal NK cells with methylation assays have demonstrated global promoter hypermethylation and gene silencing in ENKTL, with decreased mRNA transcription of 95 putative tumor suppressor genes, including *BCL2L11* (*BIM*), *DAPK1*, *PTPN6* (*SHP1*), *TET2*, *SOCS6*, and *ASNS* with known functions [26]. Specifically, *BIM* sensitizes NK cell lines to chemotherapy-induced apoptosis, *DAPK1* mediates p53-dependent apoptosis, *SHP1*, and *SOCS6* inactivate the JAK/STAT signaling pathway, and *TET2* silencing may contribute to early global hypermethylation in ENKTL, so it is easily understood how hypermethylation of multiple tumor suppressor promoters may create additive effects leading to tumor growth and malignant transformation [26]. These findings have been confirmed in other studies, with the identification of additional tumor suppressor genes, including *DLC1* involved in RAS signaling [27], *PRDM1* involved in apoptosis and promotion of NK cell growth in cooperation with activating *STAT3* mutations [28,29], *PTPRK* involved in JAK/STAT signaling and apoptosis [30], *HACE1* involved in apoptosis [31], as well as several other genes (*P73*, *hMLH1*, *CDKN2A*, *CDKN2B*, *RARβ*, *PCDH10*, *DLEC1*, *CADM*, *DAL1*) with currently unknown functions [32].

A recent genome-wide DNA methylation and transcriptomic study by Mundy-Bosse et al. (2022) demonstrated that ENKTL cells likely represent a malignant transformation from NK cells normally present in mucosal tissues, with ubiquitous and profound DNA hypermethylation driven by EBV infection causing the arrest of normal NK cell differentiation [17]. In this study, patient-derived xenograft (PDX) mouse models of ENKTL were treated with the DNA hypomethylating agent 5-azacytidine, which resulted in a significant decrease in tumor burden and an increase in OS compared to control mice. In addition, 5-azacytidine treatment resulted in a global reduction in DNA methylation and the emergence of mature NK cell markers, suggesting that treatment with hypomethylating agents may induce terminal differentiation of ENKTL cells, as is seen in patients with acute promyelocytic leukemia treated with all-trans retinoic acid [17]. These findings confirm the results of other studies, which have found that exposure of ENKL cell lines to hypomethylating agents such as 5-azacytidine or decitabine restores the expression of these putative tumor suppressors [26,27,30,146]. In a phase I study, one patient with ENKTL responded to a combination of 5-azacytidine and romidepsin with profound demethylation of their tumor after treatment [147]. There is a strong pre-clinical rationale for the treatment of ENKTL with hypomethylating agents, although there may be discordant effects from other therapeutic agents. For example, treatment with 5-azacytidine upregulates genes associated with immunoregulatory functions, suggesting possible synergy with immunotherapy agents [17], while there is concern that hypomethylating agents may desensitize ENKTL cells to asparaginase by upregulating *ASNS* and also lead to lytic reactivation of EBV [26,148].

### 3.2. Epigenetic Regulatory Genes

Previous studies of ENKTL have revealed mutations in genes that regulate epigenetic changes to the genome, including genes responsible for chromatin remodeling, histone acetyltransferases and methyltransferases, and DNA demethylases [32,149]. One of the most frequently mutated genes in 17–32% of ENKTL cases is *BCOR* [28,40,41,42], a BCL-6 interacting corepressor which is involved in the epigenetic modification of histones via HDACs and which is suggested to be a tumor suppressor given frequent loss-of-function mutations seen in ENKTL [19,41] and enhanced cell proliferation and IL-2 production when *BCOR* is silenced [42]. *MLL* genes are also commonly mutated in up to 19% of ENKTL cases, specifically *MLL2* (*KMT2D*) and *MLL3* (*KMT2C*), which are involved in histone methylation and may be tumor suppressors in B-cell lymphomas, although the role of these epigenetic regulators specifically in the tumorigenesis of ENKTL is unknown [19,28,32,43]. Other commonly mutated epigenetic regulatory genes in ENKTL include *TET1/TET2*, *EP200*, *ASXL3*, *CREBBP*, and *ARID1A*, with yet unknown functions in ENKTL biology [19,32,44].

An epigenetic regulator gene with documented oncogenic function in ENKTL is *EZH2*, a histone methyltransferase that is not mutated in ENKTL but rather is aberrantly overexpressed and promotes NK tumor cell growth independently of its histone methyltransferase activity [33]. This non-canonical activity of EZH2 is likely due to its direct phosphorylation by JAK3, which converts EZH2 from a gene repressor to a transcriptional activator of genes involved in cell proliferation [34]. The JAK/STAT pathway is likely an upstream regulator of *EZH2*, as evidenced by studies demonstrating that treatment of ENKTL cells with the JAK3 inhibitor tofacitinib reduced *EZH2* expression [38] and decreased ENKTL cell growth [34]. *EZH2* is a known druggable target, with an FDA-approved EZH2 inhibitor (tazemetostat) for EZH2-wild type and EZH2-mutated follicular lymphoma [35]. However, treatment of ENKTL PDX mice with tazemetostat alone as well as in combination with 5-azacytidine did not improve survival in one study [17] and was ineffective in ENKTL cell lines [34], suggesting that inhibition of the EZH2 methyltransferase catalytic site does not affect its non-canonical and enzyme-independent oncogenic functions. EZH2 inhibitors with novel mechanisms of action, such as 3-deazaneplanocin-A (DZNep), which disrupts the metabolism of methyl donors required for histone methylation by EZH2 and also accelerates proteasomal degradation of EZH2, have demonstrated enhanced lymphoma cell apoptosis compared to tazemetostat [36,37,38].

EZH2 inhibitors are a rational drug target in ENKTL, although there is pre-clinical evidence that combination with agents targeted at its upstream regulators, such as JAK/STAT inhibitors, may also cause ENKTL cell death in patients with high EZH2 tumor expression [34]. Further, targeting downstream target genes of EZH2, such as cyclin D1, may also be efficacious, with one study demonstrating that combined inhibition of upstream JAK with ruxolitinib and CDK4/6 with ribociclib produced synergistic inhibition of ENKTL cell growth [39].

### 3.3. Epigenetic Biomarkers

Large-scale somatic sequencing of ENKTL has enabled the description of new biomarkers which are both prognostic and predictive, although large-scale studies will be required for clinical validation. Mutations in *MLL2* (*KMT2D*) and *TET2* were associated with inferior prognosis in ENKTL [43], and overexpression of EZH2 was associated with higher tumor cell proliferation, advanced stage, and inferior survival [38]. Further, the presence of *KMT2D* mutations in circulating tumor DNA (ctDNA) was prognostic and correlated with total metabolic tumor volume, suggesting that serial measurements of ctDNA could be used to monitor treatment response and the presence of residual disease [128]. MicroRNAs (miRNAs) are short noncoding RNA sequences that inhibit the expression of target genes by suppressing translation or promoting mRNA degradation, and many have been found to be overexpressed in ENKTL as putative oncogenes as well as prognostic biomarkers [22]. For example, elevated plasma levels of miR-221 are associated with inferior overall survival [129], and miR-155 is associated with disease response [130].

Predictive biomarkers are particularly important for ENKTL to promote maximal response to therapy for patients with an aggressive and difficult-to-treat malignancy. In a study of 17 patients with ENKTL, patients with methylated *PTPRK* tumors who were treated with the SMILE protocol had significantly worse overall survival and a trend towards inferior disease-free survival [30]. In addition, *ASNS* expression is strongly correlated with cell survival in response to asparaginase treatment, which may serve as a clinically useful biomarker to determine which patients will respond to asparaginase-containing chemotherapy or which patients with low *ASNS* expression may be treated with lower doses of asparaginase to avoid toxicity [26]. miRNAs have been identified as predictive biomarkers with putative therapeutic targets; for example, a novel inhibitor of miR-155 induced apoptosis in ENKTL cell lines as well as xenografts by downregulation of STAT3 and VEGF signaling pathways and upregulation of the pro-apoptotic BRG1 [45]. High expression of SNHG12, a long noncoding RNA (lncRNAs) sequence which is overexpressed in ENKTL, conferred cisplatin resistance in ENKTL cells [131] and also may be responsible for multidrug resistance in ENKTL owing to its contribution to P-glycoprotein overexpression, which is a known mechanism for anthracycline resistance in ENTKL [150]. Another lncRNA, brain cytoplasmic RNA 1 (BCYRN1), is also overexpressed in ENKTL and is associated with inferior PFS as well as resistance to asparaginase [132].

## 4. Cell Survival and Proliferation

The second most frequently mutated genes in ENKTL NGS studies are those of the JAK/STAT signaling pathway, predominantly in ENKTL cells of NK cell lineage, while T cell lineage ENKTL more commonly exhibits mutations of the RAS/MAPK signaling pathway and aforementioned epigenetic regulators [19]. Aberrant regulation of both of these signaling pathways results in ENKTL survival and proliferation [19,149], driven by mutations in *JAK3*, *STAT3*, *STAT5B*, and *DDX3X* [19,25,149]. However, these mutations rarely occur together, suggesting that there are disparate molecular signaling pathways leading to ENKTL tumorigenesis [25] and confirmed in contemporary studies identifying three distinct subtypes of ENKTL by gene expression signatures: the TSIM (tumor suppressor-immune modulator) subtype with JAK/STAT activation and PD-L1 overexpression; the MB (MGA-BRDT) subtype with *MYC* overexpression and MAPK activation; and the HEA (HDAC9-EP300-ARID1A) subtype with changes in histone acetylation and NF-κB activation [63]. Each of these subtypes may be targeted by specific therapeutic agents based on activated signaling pathways, and putative targeted agents for each pathway are outlined as follows.

### 4.1. JAK/STAT Signaling Pathway

Constitutive activation of the JAK/STAT pathway is responsible for ENKTL survival and proliferation, with activating mutations in *STAT3* being the most common driver, followed by activating *JAK3* mutations [28,46,47,48,49]. As previously discussed, activation of the JAK/STAT pathway in ENKTL converts the histone methyltransferase EZH2 into a non-canonical transcriptional activator of genes involved in cell proliferation, with inhibition of JAK3 decreasing EZH2 expression and decreasing ENKTL tumor growth [34,38]. There are multiple inhibitors of the JAK/STAT pathway which have been studied broadly in hematologic malignancies, including specifically in ENKTL [151].

The JAK1/3 inhibitor tofacitinib has demonstrated inhibition of cell growth in JAK3-mutant [47] and STAT-3 mutant ENKTL cells [50], as well as ENKTL cells with EZH2 overexpression [34]. Similar pre-clinical results have been demonstrated with novel pan-JAK inhibitors [23] and STAT inhibitors [47,50], although none of these have yet been studied in patients. Ruxolitinib, a JAK1/2 inhibitor that is already approved for use in hematologic malignancies, is a therapy of interest in ENKTL, given pre-clinical work that JAK1/2 inhibition can disrupt JAK/STAT signaling in STAT3-mutated or overexpressed ENKTL [46]. A phase II biomarker-driven study of ruxolitinib in patients with relapsed/refractory T-cell lymphomas revealed high response rates of 45–53% in patients with activating JAK/STAT mutations or STAT3 overexpression, compared to low response rates of 13% without those biomarkers [51]. Although no patients with ENKTL were included, this study provides a strong clinical rationale for the use of ruxolitinib and other JAK/STAT inhibitors in ENKTL cases with JAK/STAT aberrations and suggests that a biomarker-based therapeutic approach utilizing the aforementioned TSIM, MB, and HEA subtypes may be warranted. Further, in vitro studies suggest that ruxolitinib may be combined with novel TP53-MDM2 inhibitors in *TP53*-wild type disease, farnesyltransferase inhibitors in *TP53-*mutated disease, and with dexamethasone or novel MCL-1 inhibitors [52], or with the BCL2 inhibitor venetoclax or aurora kinase inhibitor alisertib in another study [53], to maximize activity and avoid long-term resistance.

### 4.2. DDX3X and RAS/MAPK Signaling Pathway

*DDX3X* is an RNA helicase gene that is one of the most commonly mutated genes in ENKTL [23,28,40,64], with significant upregulation of the NF-κB and MAPK pathways found in *DDX3X*-mutated ENKTL tumors, suggesting that *DDX3X* is a possible tumor suppressor [17,19,64]. The RAS/MAPK signaling pathway is broadly implicated in carcinogenesis by promotion of cell survival and proliferation [19] and is upregulated in the MB subtype of ENKTL [63], likely related to overexpression of the EBV-associated LMP1 oncoprotein [18]. Previous pre-clinical studies have found that inhibition of the MAPK pathway is not effective in vitro or in vivo in NHL cells, likely due to the regulatory complexity of the pathway [65]. However, pre-clinical studies have found that statins inhibit cell growth and enhance chemotherapy-induced cytotoxicity in NK leukemia cell lines due to inhibition of the MAPK pathway, suggesting an opportunity for future combination studies [66]. Of note, *DDX3X* mutations portend a poor prognosis in patients with ENKTL treated with CHOP-based chemotherapy [64], although not in patients treated with more contemporary asparaginase-based regimens [63].

### 4.3. NF-κB Signaling Pathway and Survivin

There are multiple drivers of NF-κB signaling pathway upregulation in ENKTL, including *DDX3X* mutations as well as EBV-associated LMP1 oncoprotein expression [18,54]. The NF-κB pathway is considered a major driver of tumorigenesis in lymphomas and specifically in ENKTL, likely related to overexpression of a downstream anti-apoptotic protein called survivin which is elevated in nearly all cases of ENKTL and is prognostic [22,23,54,55,56,57]. There has been considerable study focused on the inhibition of the NF-κB pathway in ENKTL, particularly with the proteasome inhibitor bortezomib, which inhibits ENKTL in vitro [58]. Clinical use of bortezomib has been restricted to small case series [59], including a phase II study of bortezomib, gemcitabine, ifosfamide, and oxaliplatin in 7 patients with newly-diagnosed ENKTL which demonstrated an overall response rate of 43% but a median progression-free survival of only 4 months [60]. A phase II study of bortezomib in combination with CHOP chemotherapy included 10 patients with ENKTL, for which the complete response rate was much lower than other lymphoma subtypes at 30%, with ENKTL patients having rapid disease progression after the first two cycles, likely due to known anthracycline resistance in ENKTL [61]. There is a suggestion that bortezomib may be effective if combined with asparaginase-based chemotherapy, as inhibition of the NF-κB pathway would downregulate CD25, which induces asparaginase resistance in ENKTL cell lines [23], and effective combinations of proteasome inhibitors with other chemotherapeutics or targeted agents merits further study. For example, pre-clinical data has identified that terameprocol and mithramycin, inhibitors of survivin and its related transcription factors, induce apoptosis of ENKTL cells, with mithramycin also suppressing ENKTL growth in a mouse xenograft model, both of which may be appropriate partners for multipronged inhibition of the NK-κB pathway [55,62].

### 4.4. C-MYC

*C-MYC* is a well-known oncogene in lymphomas and is overexpressed in ENKTL, likely driven by LMP1 and EBV infection and causing upregulation of downstream targets *EZH2* and *RUNX3*, the latter of which is a master transcriptional regulator and a putative oncogene in ENKTL [22,67,68]. *C-MYC* inhibitors are of particular interest in multiple aggressive lymphomas, and treatment of ENKTL cell lines with a small-molecule novel *MYC* inhibitor caused downregulation of both *MYC* and *RUNX3* and, subsequently, apoptosis [67].

### 4.5. PDGFRa

Platelet-derived growth factor receptor alpha (PDGFRa) is a tyrosine kinase pathway that is overexpressed in ENKTL, possibly driven by the PI3K/Akt/mTOR and JAK/STAT pathways, and mediates cell survival [69,70]. Previous pre-clinical work has demonstrated that imatinib, a tyrosine kinase inhibitor (TKI), inhibits ENKTL cell proliferation in vitro [71] as well as in mouse models [69], supporting further study of TKIs in ENKTL. In addition, high PDGFRa expression is a prognostic biomarker of survival [69,70].

### 4.6. PI3K/Akt/mTOR Signaling Pathway

The PI3K/Akt/mTOR signaling pathway, which is a master regulator of growth and survival in normal and malignant cells, is downstream of the JAK/STAT pathway and is upregulated in many types of lymphoma, including ENKTL, where it is driven by EBV infection and LMP1 [18,23,72]. Inhibitors of multiple targets along this signaling cascade have been explored in lymphomas, particularly PI3K inhibitors, which have already been approved by the FDA for various relapsed/refractory lymphoma subtypes. Several small phase I studies of duvelisib and tenalisib in peripheral T-cell lymphomas (PTCLs) have demonstrated an ORR of ~50% [73,74,75], and a phase I/II trial of copanlisib and gemcitabine in PTCL included 3 patients with ENKTL with 2 partial responses and 1 complete response [76]. Combination targeting of the PI3K/Akt/mTOR signaling pathway appears to be more effective due to the activation of alternative signaling pathways by negative feedback, with drug sensitivity profiling revealing that inhibition of both mTOR and JAK may be more effective than single agents in NK/T-cell lymphoma cell lines [77]. As an example, a phase I/II trial of the mTOR inhibitor temsirolimus in combination with the Akt inhibitor and immunomodulatory agent lenalidomide in relapsed/refractory lymphomas demonstrated an ORR of 67% in 9 patients with T-cell lymphomas, a superior ORR compared to single-agent mTOR inhibition or lenalidomide alone [78].

## 5. Avoiding Autophagy

Avoiding apoptosis, either via overexpression of anti-apoptotic proteins or suppression of tumor suppressor genes, has been identified as a significant contributor to tumorigenesis in ENKTL. The tumor suppressor *TP53* is one of the most frequently mutated genes in ENKTL and is present in up to 63% of cases [19,28,40,64], with a worse prognosis in *TP53*-mutated ENKTL [133]. Although genomic studies have identified numerous other putative tumor suppressor genes in ENKTL with functional suppression of cell growth in ENKTL cell lines, such as *PRDM1* and *FOXO3*, there are not currently targeted agents to re-enable these tumor suppressors [152]. However, histone deacetylase inhibitors have been explored extensively in lymphomas to reactivate intrinsic apoptotic pathways, with several HDAC inhibitor agents under exploration in ENKTL [79].

### Histone Acetylation and HDAC Inhibitors

As previously discussed, HDACs are histone deacetylases that frequently lead to the inactivation of tumor suppressor genes when aberrantly activated during tumorigenesis [32]. As such, HDAC inhibitors lead to the reactivation of downstream apoptotic pathways, including upregulation of *TP53* and downregulation of the PI3K/Akt/mTOR pathway by the accumulation of autophagic factors [79]. Given the known activity of the HDAC inhibitor romidepsin in T-cell lymphomas, a prospective pilot study of romidepsin, specifically patients with relapsed/refractory ENKTL, was conducted in 2013 [80]. However, of the first 5 patients enrolled in the study, 3 patients developed treatment-emergent EBV reactivation manifesting as severe liver injury and 1 death, resulting in early termination of the trial. A correlative analysis confirmed that romidepsin caused a significantly greater increase in EBV reactivation in ENKTL cell lines compared to vorinostat [80]. This early experience decreased interest in HDAC inhibitors for ENKTL, although there was a suggestion that not all HDAC inhibitors would cause the same degree of EBV reactivation given different HDAC isoform specificity of different inhibitors. Subsequent clinical studies have explored various HDAC inhibitors in ENKTL, including chidamide, which is of particular interest given it also inhibits the PI3K/Akt/mTOR and RAS/MAPK signaling pathways, with an ORR of 15–50% in small studies of 15–19 patients [81,82,83]. There have also been responses in single patients to belinostat [84] and vorinostat [85]. Simultaneous targeting of the NF-κB signaling pathway with proteasome inhibitors was posited to reduce the risk of EBV reactivation in a phase II study of panobinostat and bortezomib, in which 1 of 2 patients with ENKTL had a partial response, and neither patient had EBV reactivation [153,154].

Several other HDAC inhibitor combinations have been explored, including chidamide in combination with chemotherapy as well as chidamide in combination with immunotherapy, the latter to be discussed later. In the former study of 53 patients with ENKTL, the ORR was significantly higher when chidamide was combined with chemotherapy (40%) compared to chidamide alone (15%), confirming pre-clinical data that HDAC inhibitors sensitize lymphoma cells to chemotherapy-induced DNA damage [82]. In a phase II randomized trial of 74 patients with newly-diagnosed, high-risk, early-stage ENKTL, patients were treated with radiation therapy followed by gemcitabine, dexamethasone, and cisplatin (GDP) either with or without chidamide [155]. Response rates, PFS, and OS were similar in the control group of GDP alone as compared to the experimental arm of GDP combined with chidamide, suggesting that chidamide may need to be paired with a different chemotherapy backbone or studied in patients with specific biomarkers such as the HEA subtype of ENKTL which is enriched with histone acetylation mutations [63]. In that vein, a phase II single-arm trial of 36 patients with ENKTL in first complete response were administered chidamide, cladribine, gemcitabine, and busulfan conditioning chemotherapy prior to autologous stem cell transplantation [156]. The 4-year PFS of ENKTL patients was 73%, which was significantly higher than historical PFS rates of 33–40% after autologous transplantation and suggested that the optimum pairing of HDAC inhibitors with chemotherapy partners merits further study. Novel HDAC inhibitors with different mechanisms of action have also been developed, including nanatinostat in combination with valganciclovir [122], citarinostat in combination with the JAK/STAT inhibitor momelotinib in cell lines [157], and the histone acetyltransferase KAT5 inhibitor NU9056 in cell lines [158].

## 6. Tumor Microenvironment

Mobilizing the tumor microenvironment (TME) is a major therapeutic advancement in cancer, particularly with checkpoint inhibitors which augment the PD-1/PD-L1 signaling pathway to induce a cytotoxic T cell immune response against tumor cells. In lymphomas, immunotherapy has been most successful in Hodgkin lymphoma, in which anti-PD1 treatment alters the TME by downregulating pro-survival factors rather than inducing an immune response [159]. The role of the TME in ENKTL is under investigation, with previous studies identifying the upregulation of PD-L1 in ENKTL cells driven by EBV-mediated LMP1 and constitutive activation of the JAK/STAT pathway [134,160]. However, there are additional elements of the TME in ENKTL that may be amenable to therapeutic targeting.

### 6.1. Immune Surveillance and the PD-1/PD-L1 Signaling Pathway

PD-L1 is very commonly expressed in ENTKL in 56–80% of cases [86,87], with the interplay of PD-1 and PD-L1 in the TME of particular interest in the therapeutic targeting of ENKTL, with a study finding that PD-L1 is expressed on both tumor cells as well as tumor-infiltrating macrophages in the ENKTL TME, suggesting that ENKTL cells may additionally induce immunosuppression by upregulation of PD-L1 in macrophages [88]. Commercial monoclonal antibodies against PD-1, such as pembrolizumab and nivolumab, have been studied in small cohorts of patients with relapsed/refractory ENKTL: ORR 100% in 7 patients using various radiographic and serologic response criteria [89], ORR 57% in 7 patients [90] and in 2 out of 3 patients of whom 2 quickly died due to infections and poor performance status [91], as well as in case reports [92,93]. However, the follow-up in these studies was very short at less than 6 months, with PFS and OS also measured in less than 6 months for most of the patients. Sintilimab, an anti-PD-1 antibody with a different binding site than pembrolizumab and nivolumab with greater affinity for PD-1, has been extensively studied in ENKTL. In an initial phase II study of sintilimab monotherapy in 28 patients with relapsed/refractory ENKTL, the ORR was 75% (CR 21%) with a 2-year OS of 79% with 30 months of median follow-up [94]. This trial generated significant interest in sintilimab, given high survival rates in patients with an otherwise dismal prognosis, and also demonstrated that patients with pseudo-progression due to immunotherapy, which developed in 18% of patients in the study, did not have an inferior survival and benefitted from ongoing continuous treatment with sintilimab.

Given the low complete response rates with sintilimab monotherapy, there have been several efforts to combine immunotherapy with chemotherapy and other agents to induce deeper and prolonged responses, including in the frontline setting. The initial experience combining various anti-PD-1 agents with peg-asparaginase, gemcitabine, and oxaliplatin (P-GemOx) in 9 patients with newly-diagnosed, advanced-stage ENKTL revealed an ORR of 89% (78% CR) with 1-year PFS and OS of 67% and 100%, respectively, with follow-up of 10.6 months [95]. This led to a subsequent phase II study of sintilimab plus P-GemOx, with preliminary results of 6 patients revealing an ORR of 100% (33% CR) with only 7.8 months of follow-up [96]. The short follow-up of these studies does not confirm if combination therapy improves the promising survival observed with sintilimab monotherapy, although a stage-adapted study of sintilimab plus chidamide induction followed by P-GemOx (plus radiation for early-stage disease) demonstrated an ORR of 100% for 19 patients with early-stage disease and 100% in 7 out of 9 patients with advanced-stage disease who were able to complete therapy [97]. The 1-year PFS and OS rates were both 93% across the entire cohort, demonstrating that a non-chemotherapy pre-phase may be effective in patients with early-stage disease who present with poor performance status. However, given that the ORR was 100% after sintilimab and chidamide induction in the early-stage cohort and only 44% in the advanced-stage group, with 3 patients developing rapid progression during induction in the latter cohort, the appropriate choice and sequence of combination therapies need to be carefully selected based on stage as well as predictive biomarkers. The combination of sintilimab plus chidamide also has activity in the relapsed/refractory setting, with an ORR of 60% (CR 49%) and an 18-month OS of 76% in 38 patients [98]. Other combinations have been explored, including anti-PD-1 tislelizumab with chidamide, lenalidomide, and etoposide in 8 patients (ORR 88%, CR 63%) [99], anti-PD-1 toripalimab with chidamide, etoposide, and thalidomide in 3 patients (ORR 100%, CR 67%) [100], case reports of sintilimab [101] and atezolizumab [102] with chidamide, and ongoing study of the hypomethylating agent azacitidine with tislelizumab and peg-asparaginase [103]. A more recent 2022 abstract reported the results of 27 patients with relapsed and refractory ENKTL who were treated with various anti-PD-1 antibodies in conjunction with hypomethylating agents (either decitabine or azacytidine), finding an ORR of 63% (CR 59%), including high ORRs in patients who had previously received checkpoint inhibitors, with median PFS of 12.8 months [104].

Sintilimab is not currently available in the United States, although there are other novel immunotherapy agents which are amenable to study, such as the anti-PD-L1 agent sugemalimab which received breakthrough therapy designation by the FDA for relapsed/refractory ENKTL in 2020 based on the GEMSTONE-201 trial, which preliminarily demonstrated an ORR of 46% (CR 37%) with 2-year OS of 55% in 80 patients with relapsed/refractory ENKTL [105,106]. The anti-PD-L1 agent avelumab has activity in relapsed/refractory disease, with an ORR of 38% (CR 24%) among 21 patients, with expression of PD-L1 significantly associated with complete responses [107]. A novel recombinant anti-PD-1 agent, geptanolimab, was studied in 102 patients with relapsed/refractory PTCL, with the highest ORR of 63% among the 19 patients with ENKTL. There are also novel agents under investigation, including the anti-PD-1 IgG4 antibody camrelizumab [161], as well as monoclonal antibodies for newly-described immune checkpoint receptors in NK cell lymphomas such as killer cell lectin-like receptor G1 (KLRG1) [162].

The available data suggest that immunotherapy is a rational and efficacious treatment for both newly-diagnosed and relapsed/refractory ENKTL, although long-term follow-up is necessary to assess if responses as monotherapy or combination therapy are durable. There have been mixed results in ENKTL with regards to the prognostic value of PD-L1 in ENKTL, with one study finding that higher expression of PD-L1 expression was associated with inferior treatment response and survival in early-stage disease when defined as ≥38% on histology [134], but improved survival in advanced-stage disease when defined as ≥10% on histology [86]. Given emerging data about different molecular subtypes of ENKTL, it is possible that a biomarker-driven approach may be warranted, with immunotherapy targeted towards patients with the TSIM subtype characterized by PD-L1 overexpression, or patients with mutated *PD-L1* who have better responses to pembrolizumab [135]. Recent whole-genome sequencing has further identified 4 TME subgroups of ENKTL: an ‘immune tolerance’ group with high numbers of Tregs more common in early-stage disease; two ‘immune evasion’ groups with frequent cytotoxic T cells and high PD-L1 expression; and an ‘immune silenced’ group with an exhausted immune response more common in advanced-stage disease [136]. The ‘immune silenced’ group had the worst prognosis and also had the worst response to pembrolizumab, with the best responses seen in the ‘immune tolerance’ group. These data suggest that immunotherapy may be targeted towards patients with the aforementioned predictive biomarkers or potentially to patients with early-stage disease who are more likely to have immunotherapy-responsive phenotypes.

### 6.2. Angiogenesis

Several important molecular drivers of angiogenesis are upregulated in ENKTL, including vascular endothelial growth factor (VEGF) and its genes (*VEGFA*, *VEGFC*, and *VEGFR2*), as well as the oncogene *MET* and its related hepatocyte growth factor (HGF), all of which are regulated by the JAK/STAT pathway [71]. Anti-VEGF antibodies such as bevacizumab have been studied in lymphomas, including a study of bevacizumab plus CHOP in 39 patients with T and NK cell lymphomas with an ORR of 90% (CR 49%) which demonstrated significant treatment-limiting toxicities and poor durability [108]. However, a phase II trial of bevacizumab, gemcitabine, peg-asparaginase, oxaliplatin, and dexamethasone in 43 patients with newly-diagnosed ENKTL demonstrated an ORR of 100% (CR 100% for early-stage and 88% for advanced-stage disease) with a 2-year PFS and OS of 83% and 79%, respectively [109]. An oral tyrosine kinase inhibitor specific for the VEGF receptor VEGFR2, apatinib, is being studied in conjunction with the anti-PD-1 inhibitor camrelizumab in relapsed/refractory PTCL, with preliminary results demonstrating that 2 of the 3 patients with ENKTL achieved a partial response [110]. Pre-clinical data also support the use of *MET* inhibitors, which induce enhanced helper T cell recognition of ENKTL cell lines and may be combined with immunotherapy [111], and oral MET inhibitors such as crizotinib have already been studied in other types of PTCL with an ORR of 90% in ALK-positive lymphomas [112]. Serum VEGF levels are prognostic of PFS and OS in patients with ENKTL treated with non-anthracycline-based chemotherapy [56].

### 6.3. Cytokines and Chemokines

The inflammatory milieu of the ENKTL TME is thought to contribute to lymphomagenesis, as EBV infection causes NK/T cells to become sensitized to growth-promoting cytokines such as IL-2 [24]. Several serum cytokines are prognostic in ENKTL, including IL-18, which is associated with HLH, advanced-stage disease, and poor OS [137]; IL-10, which is associated with worse OS, low CR rate, and higher early relapse rate [138]; and IL-2Ra, IL-9, IL-15, and MIP-1a [139]. In addition, interferon, IL-6, and IL-10 are all associated with serum EBV DNA load, a known prognostic biomarker in ENKTL, suggesting that EBV-induced cytokine storm may contribute to poor prognosis in ENKTL [140,141]. Inhibition of proliferative cytokines has been proposed as a treatment strategy in ENKTL, although there is no clinical data. However, a recent study suggests that IL-10 contributes to gemcitabine resistance in ENKTL cell lines by regulation of drug resistance genes, suggesting that inhibition of IL-10 in select patients may be utilized to increase tumor sensitivity to chemotherapy [113].

Chemokines are chemoattractant cytokines that also mediate the TME in EBV-driven malignancies and promote metastasis and tumor angiogenesis, with chemokine receptor 4 (CCR4), the receptor for chemokine ligand 17 and 22 (CCL17 and CCL22), prominently identified in ENKTL tumors [114]. Mogamulizumab, a monoclonal antibody against CCR4, has demonstrated cytotoxicity in ENKTL cell lines and has clinical activity in other T cell malignancies, warranting further study [114].

### 6.4. S100A9

Recent proteomic analysis has identified a novel biomarker in ENKTL, S100A9, an immunosuppressive molecule that is overexpressed in serum and tumor samples in ENKTL and likely mediates tumorigenesis by upregulation of PD-L1 expression [115]. High levels of S100A9 are associated with poor response to therapy, early relapse, and advanced stage, and it has been proposed as a possible therapeutic target.

## 7. Other Therapeutic Targets

Given the abysmal prognosis of relapsed and refractory ENKTL, numerous targeted agents have been trialed in single patients or small cohorts with variable efficacy. However, there is pre-clinical rationale for the combination of these targeted agents with other active chemotherapeutics in ENKTL.

### 7.1. CD38

Cell surface protein CD38 is expressed in the vast majority of ENKTL cases, and high expression of CD38 is correlated with inferior responses and survival [116]. The anti-CD38 monoclonal antibody daratumumab has been explored in relapsed/refractory ENKTL in a phase II study of 32 patients, which demonstrated an ORR of 25% (no CRs) with a very short response duration, with a median duration of response of 55 days and a 6-month OS of 43% [117]. Although daratumumab has low single-agent activity, it is frequently used in combination with other therapies in multiple myeloma, and there is emerging pre-clinical evidence that daratumumab enhances the cytotoxicity of asparaginase in a PDX mouse model; in that same study, pretreatment of ENKTL cell lines with all-trans retinoic acid (ATRA) increased expression of CD38 and increased daratumumab-induced cytotoxicity, setting the stage for future combination studies [118].

### 7.2. CD30

The cell surface protein CD30 is also expressed in the majority of ENKTL cases [119], with an unclear role as a prognostic marker [142,143]. Brentuximab vedotin (BV), a CD30-directed antibody-drug conjugate that is used extensively in lymphomas, has low single-agent activity in relapsed/refractory ENKTL, with a phase II study that included 7 patients with ENKTL demonstrating an ORR of 29% (1 patient with CR) [120]. P-glycoprotein (MDR1) may mediate resistance to BV in ENKTL, and cyclosporine, an MDR1 inhibitor, may increase BV response rates [121]. There are ongoing studies to combine BV with combination chemoimmunotherapy as is done in other lymphomas, as well as CD30-directed CAR T cell therapy which may provide therapeutic avenues [23,142].

### 7.3. EBV-Targeted Approaches

Targeting EBV and its myriad viral proteins which drive ENKTL tumorigenesis, including LMP1 and LMP2, have been explored [123], particularly the use of antigen-specific cytotoxic T-lymphocytes (CTLs) as has been utilized in EBV-associated PTLD. In one study, LMP-targeted autologous CTLs were infused into patients with EBV-associated lymphomas, including 11 patients with ENKTL [124]. The ORR was 67% (all CRs) in the 6 patients with active disease, with durable remissions of 4 or more years in 3 of the patients and no treatment-emergent toxicities. In the 5 patients who were already in remission and felt to be at high risk of relapse, all 5 patients had ongoing sustained remission after CTL infusion with no CTL-related toxicities, suggesting that consolidation with CTLs may also be a therapeutic approach. This has been demonstrated in a cohort of 26 patients who had undergone allogeneic transplantation for EBV-associated lymphomas and who adjuvantly received allogeneic donor-derived LMP-specific T-cells, which demonstrated a 2-year EFS of 57% [125]. A similar study of adjuvant CTLs in 10 patients with ENKTL who were in a CR after induction therapy demonstrated a 4-year PFS and OS of 90% and 100%, respectively, suggesting that the post-remission timepoint may be a critical period to consolidate with CTL therapy [126].

A more recent study explored the activity of a novel T cell product, baltaleucel-T, in which autologous peripheral T cells are stimulated with antigen-presenting cells containing peptide libraries of multiple EBV-associated proteins, including LMP1, LMP2, and multiple cytokines [127]. Of the 54 patients with advanced-stage, relapsed ENKTL screened for the study, only 47 had adequate whole blood collection, and only 15 patients actually received baltaleucel-T treatment, with the other patients either having manufacturing failure and death or disease progression death before product administration. The ORR among 10 patients with measurable disease was 50% (CR 30%), with a median PFS of 12 months. Although there was activity of the product in relapsed ENKTL, manufacturing issues present a significant challenge in an aggressive and rapidly-progressive malignancy, and off-the-shelf allogeneic products are under investigation.

Antiviral therapy against EBV requires the presence of lytic phase viral proteins, and HDAC inhibitors are able to activate the lytic cycle to sensitize EBV-infected cells to ganciclovir. A phase I/II study of nanatinostat with valganciclovir in 55 patients with relapsed/refractory EBV-positive lymphomas included 9 patients with ENKTL and demonstrated an ORR of 60% (27% CR) in all patients with T/NK cell lymphomas and an ORR of 63% in ENKTL patients [122].

## 8. Conclusions and Future Directions

Next-generation sequencing and proteomic analyses over the past decade have significantly improved our understanding of the molecular landscape that induces ENKTL tumorigenesis [23,28] and have revealed several novel therapeutic approaches. Given that EBV-driven DNA hypermethylation is ubiquitously observed in ENKTL, combinations of hypomethylating agents with other therapeutic agents, particularly immunotherapy, in which synergy has already been demonstrated, present promising opportunities for future clinical trials [17,104]. These innovations have also revealed new predictive biomarkers in newly-diagnosed and relapsed disease as well as refined prognostic and predictive scoring systems, including composite single-nucleotide polymorphism signatures [144] and identification of unique genetic clusters of ENKTL [28,145], which may enable a personalized medicine approach towards ENKTL therapy. Prospective trials which incorporate these biomarkers and novel therapeutics in rational combinations with existing standard-of-care treatment regimens will be vital to validate their clinical utility and new treatment paradigms for this aggressive malignancy.

## Figures and Tables

**Table 1:** Putative driver mutations in ENKTL and potential therapeutic approaches.

Identified Mutation and Prevalence (If Available)	Biologic Significance in ENKTL	Hypothetical Therapeutic Approach	References
Promoter hypermethylation			
*BCL2L11* (*BIM*)	sensitizes NK cell lines to chemotherapy-induced apoptosis	hypomethylating agents (azacitidine, decitabine)	[26]
*DAPK1*	mediates p53-dependent apoptosis		[26]
*PTPN6* (*SHP1*)	inactivates the JAK/STAT signaling pathway		[26]
*TET2* 9%	contributes to early global hypermethylation		[26]
*SOCS6*	inactivates the JAK/STAT signaling pathway		[26]
*ASNS*	downregulation is associated with increased sensitivity to L-asparaginase		[26]
*DLC1*	RAS signaling		[27]
*PRDM1* 6%	apoptosis		[28,29]
*PTPRK* 10%	JAK/STAT signaling and apoptosis		[30]
*HACE1*	apoptosis		[31]
* P73 * , *hMLH1*, *CDKN2A*, *CDKN2B*, *RARβ*, *PCDH10*, *DLEC1*, *CADM*, *DAL1*	currently unknown functions		[32]
Epigenetic regulatory genes			
*EZH2* 61%	aberrant overexpression results in tumor growth independent of its histone methyltransferase activity via non-canonical transcriptional activator	3-deazaneplanocin-A (DZNep) tazemetostat (minimal activity in ENKTL) CDK4/6 inhibitors (i.e., ribociclib) for downstream cyclin D1 inhibition	[33,34,35,36,37,38,39]
* BCOR * 17–32%	tumor suppressor: suppression leads to enhanced cell proliferation and IL-2 production via HDACs	none	[19,28,40,41,42]
* MLL2 * (*KMT2D*) and *MLL3* (*KMT2C*) 13–19%	tumor suppressor related to histone methylation	none	[19,28,32,43]
*TET1/TET2*, *EP200*, *ASXL3*, *CREBBP* and *ARID1A* 5–9%	currently unknown functions	none	[19,32,44]
MicroRNAs (i.e., miR-155)	miR-155 activates STAT3 and VEGF signaling to promote lymphomagenesis	novel miRNA inhibitors (i.e., for mIR-155)	[22,45]
Cell survival and proliferation			
JAK/STAT pathway (usually *STAT3* or *JAK3* mutations) 18%	constitutive activation and downstream EZH2 upregulation	tofacitinib ruxolitinib (combined with novel TP53-MDM2, farnesyltransferase, or MCL-1 inhibitors, or with BCL2 inhibitor venetoclax or aurora kinase inhibitor alisertib) novel pan-JAK and STAT inhibitors momelotinib (combined with HDAC inhibitor citarinostat)	[23,28,34,38,46,47,48,49,50,51,52,53]
NF-κB signaling pathway and survivin 7%	constitutive activation and downstream upregulation of anti-apoptotic protein survivin	proteasome inhibitors (bortezomib, carfilzomib) survivin inhibitors (terameprocol, mithramycin)	[22,23,54,55,56,57,58,59,60,61,62]
*DDX3X* and RAS/MAPK pathway 14%	tumor suppressor: upregulation of downstream NF-κB and MAPK pathways, resulted in cell proliferation	previous inhibitors of MAPK pathway not effective statins (i.e., fluvastatin, atorvastatin)	[17,18,19,23,28,40,63,64,65,66]
*C-MYC* 45%	oncogene causing downstream upregulation of *EZH2* and *RUNX3*	novel small molecule *MYC* inhibitors (i.e., JQ1)	[22,67,68]
PDGFRa pathway 89–91%	upregulated by PI3K/Akt/mTOR and JAK/STAT pathways and mediates cell survival via tyrosine kinase activity	tyrosine kinase inhibitors (i.e., imatinib)	[69,70,71]
PI3K/Akt/mTOR pathway	upregulated by JAK/STAT pathway and mediates cell survival	PI3K inhibitors (i.e., duvelisib) Akt inhibitor lenalidomide mTOR inhibitors (i.e., temsirolimus) may be more effective in combination	[18,23,72,73,74,75,76,77,78]
Avoiding autophagy			
HDACs	inactivation of tumor suppressor genes	HDAC inhibitors (i.e., romidepsin, vorinostat, chidamide, panobinostat, nantinostat, citarinostat) novel histone acetyltransferase inhibitors (KAT5 inhibitor NU9056)	[32,79,80,81,82,83,84,85]
*TP53* (63%), *PRDM1*, *FOXO3*	tumor suppressors	none (putative inhibitors for *TP53-*mutated and *TP53*-wild type disease)	[19,28,40,52,64]
Tumor microenvironment			
PD-1/PD-L1 pathway 56–80%	PD-L1 overexpression on tumor cells and tumor-infiltrating macrophages enabling escape from immune surveillance	PD-1 inhibitors (pembrolizumab, nivolumab, sintilimab, tislelizumab, toripalimab, geptanolimab, camrelizumab) PD-L1 inhibitors (atezolizumab, sugemalimab, avelumab)	[86,87,88,89,90,91,92,93,94,95,96,97,98,99,100,101,102,103,104,105,106,107]
VEGF	overexpression and tumor angiogenesis	anti-VEGF monoclonal antibodies (i.e., bevacizumab) VEGF receptor inhibitor apatinib	[71,108,109,110]
*MET* and HGF	overexpression and tumor angiogenesis, invasion, and metastasis	*MET* inhibitors (i.e., crizotinib)	[71,111,112]
Cytokines (IL-2, IL-18, IL-10, IL-9, IL-15, MIP-1a, IL-6, interferon)	growth promotion	cytokine inhibitors (not currently being studied in ENKTL)	[24,113]
Chemokines (CCR4)	promote tumor angiogenesis and metastasis	mogamulizumab	[114]
* S100A9 *	upregulates PD-L1 expression and escape from immune surveillance	none	[115]
Other therapeutic targets			
CD38 50%	cell surface binding mediates cell adhesion and proliferation of lymphocytes	daratumumab (ATRA may enhance daratumumab-induced cytotoxicity)	[116,117,118]
CD30 57%	cell surface binding stimulates the NF-κB and MAPK pathways with pro-survival and anti-apoptotic effects	brentuximab vedotin	[119,120,121]
EBV	overexpression of the oncoprotein LMP1, leading to ligand-independent activation of the pro-survival and anti-apoptotic NF-κB and MAPK pathways	autologous or allogeneic cytotoxic T-lymphocyte cellular therapies (i.e., baltaleucel-T) antiviral therapy (valganciclovir, in combination with HDAC inhibitors)	[122,123,124,125,126,127]

**Table 2:** Prognostic and predictive biomarkers in ENKTL.

Identified Mutation	Prognostic or Predictive	Significance in ENKTL	References
*MLL2* (*KMT2D*)	prognostic	mutation associated with inferior overall survival mutation in circulating ctDNA associated with total metabolic tumor volume	[43,128]
*TET2*	prognostic	mutation associated with inferior overall survival	[43]
EZH2	prognostic	overexpression associated with higher tumor cell proliferation, advanced stage, and inferior overall survival	[38]
MicroRNAs (i.e., miR-221 and miR-155)	prognostic	high serum miR-221 associated with inferior overall survival high serum miR-155 associated with disease response	[22,129,130]
*PTPRK*	predictive	tumors with methylated *PTPRK* promoter regions treated with SMILE protocol had an inferior overall survival	[30]
*ASNS*	predictive	high expression associated with asparaginase resistance	[26]
lnRNAs (i.e., SNHG12 and BCYRN1)	prognostic predictive	high SNHG12 expression associated with cisplatin resistance as well as multidrug resistance via P-glycoprotein high BCYRN1 expression associated with inferior PFS and resistance to asparaginase	[131,132]
Gene expression signatures	prognostic predictive	three molecular subtypes of ENKTL: tumor-suppressor/immune-modulator (TSIM), MYC-related (MB) and histone epigenetic altered (HEA) superior survival in HEA subtype and inferior survival in MB subtype TSIM subtype predicts response to checkpoint inhibitors, MB subtype predicts response to MYC inhibitors, and HEA subtype predicts response to HDAC inhibitors	[63]
JAK/STAT	predictive	activating JAK/STAT mutations or STAT3 overexpression predicts response to ruxolitinib	[51]
*TP53*	prognostic predictive	TP53 associated with inferior survival mutational status predicts response to agents in combination with ruxolitinib: novel TP53-MDM2 inhibitors in TP53-wild type disease and farnesyltransferase inhibitors in TP53-mutated disease	[52,133]
*DDX3X*	prognostic	mutation associated with inferior prognosis and inferior response to CHOP-based chemotherapy but not asparaginase-based chemotherapy	[63,64]
survivin	prognostic	overexpression associated with inferior survival	[22,23,54,55,56,57]
*C-MYC*	prognostic	presence of C-MYC associated with inferior overall survival	[22,63,67,68]
PDGFRa	prognostic	high expression associated with inferior PFS	[69,70]
PD-L1	prognostic predictive	mixed results: high expression associated with inferior treatment response and survival in early-stage disease but improved survival in advanced-stage disease mutated *PD-L1* gene is associated with better responses to pembrolizumab	[63,86,134,135]
Whole genome sequencing	prognostic predictive	four immune subgroups of ENKTL: an ‘immune tolerance’ group (high numbers of Tregs, early stage, best prognosis); two ‘immune evasion’ groups (high cytotoxic T-cells and high PD-L1); and an ‘immune silenced’ group (exhausted immune response, advanced stage, worst prognosis) ‘immune silenced’ group had the worse response to pembrolizumab; ‘immune tolerance’ group had the best response to pembrolizumab	[136]
VEGF	prognostic	high serum VEGF levels associated with inferior PFS and OS	[56]
Serum cytokines	prognostic predictive	high serum IL-18, IL-10, IL-6, IL-2Ra, IL-9, IL-15, MIP-1a all associated with inferior survival high serum IL-10 predicts resistance in gemcitabine	[113,137,138,139,140,141]
S100A9	prognostic	high serum and tumor levels associated with advanced stage, poor response to therapy, and early relapse	[115]
CD38	prognostic predictive	high expression associated with inferior survival predicts response to daratumumab	[116]
CD30	prognostic predictive	mixed results with unclear effect on prognosis predicts response to brentuximab vedotin	[142,143]
single-nucleotide polymorphisms (SNPs)	prognostic	composite signature of 7 SNPs predicts PFS and OS	[144]
genome-wide mutation and genomic copy number alterations (gCNAs) analysis	prognostic	seven distinct genetic clusters of ENKTL identified with differences in overall survival between clusters	[145]

## Data Availability

All data can be found in the text.
